# A single-source precursor approach to solution processed indium arsenide thin films†
†Electronic supplementary information (ESI) available: Table listing selected bond lengths and angles for InAs precursor complex. Cross-sectional SEM of InAs thin film. XPS depth profile spectra of InAs thin film. Valence band XPS of InAs thin film and standard. CCDC 1477895. For ESI and crystallographic data in CIF or other electronic format see DOI: 10.1039/c6tc02293f



**DOI:** 10.1039/c6tc02293f

**Published:** 2016-06-23

**Authors:** Peter Marchand, Sanjayan Sathasivam, Benjamin A. D. Williamson, David Pugh, Salem M. Bawaked, Sulaiman N. Basahel, Abdullah Y. Obaid, David O. Scanlon, Ivan P. Parkin, Claire J. Carmalt

**Affiliations:** a Materials Chemistry Centre , Department of Chemistry , University College London , 20 Gordon Street , London WC1H 0AJ , UK . Email: c.j.carmalt@ucl.ac.uk; b Bio Nano Consulting Ltd , The Gridiron Building , One Pancras Square , London N1C 4AG , UK; c Kathleen Lonsdale Materials Chemistry , Department of Chemistry , University College London , 20 Gordon Street , London WC1H 0AJ , UK; d Department of Chemistry , Imperial College London , South Kensington , London , SW7 2AZ , UK; e Chemistry Department , Faculty of Science , King Abdulaziz University , Saudi Arabia; f Surface Chemistry and Catalytic Studies Group , King Abdulaziz University , Saudi Arabia; g Diamond Light Source Ltd. , Diamond House , Harwell Science and Innovation Campus , Didcot , Oxfordshire OX11 0DE , UK

## Abstract

This paper reports the synthesis of the novel single-source precursor, [{(MeInAs^*t*^Bu)_3_}_2_(Me_2_InAs(^*t*^Bu)H)_2_] and the subsequent first report of aerosol-assisted chemical vapour deposition of InAs thin films. Owing to the use of the single-source precursor, highly crystalline and stoichiometric films were grown at a relatively low deposition temperature of 450 °C. Core level XPS depth profiling studies showed some partial oxidation of the film surface, however this was self-limiting and disappeared on etch profiles. Valence band XPS analysis matched well with the simulated density of state spectrum. Hall effect measurements performed on the films showed that the films were n-type with promising resistivity (3.6 × 10^–3^ Ω cm) and carrier mobility (410 cm^2^ V^–1^ s^–1^) values despite growth on amorphous glass substrates.

## Abstract 2

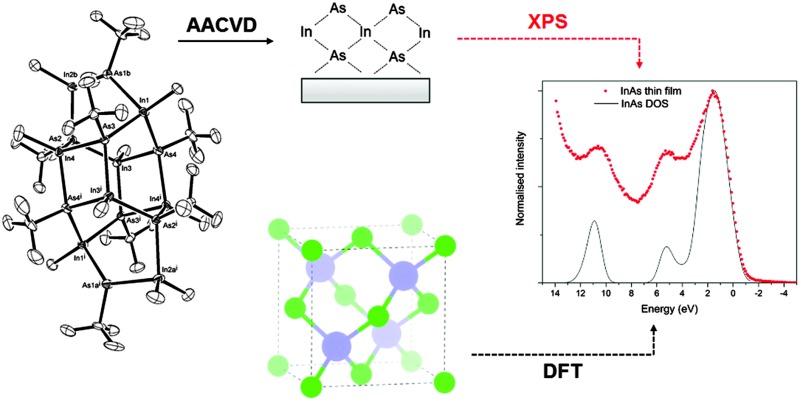
Highly crystalline, stoichiometric InAs films have been deposited utilising a novel InAs cluster as a single source precursor.

## Introduction

The III/V compounds of group 13 metals aluminium, gallium and indium are an important class of semiconductor materials for technological applications. In comparison to the isoelectronic elemental group 14 materials (silicon, germanium), III/V semiconductors typically offer high electron mobilities and direct band gaps (excluding GaP and AlP), giving them particular use in optoelectronic devices such as light emitting diodes (LEDs).^1,2^ InAs has a direct band gap of 0.35 eV and has been known to display high electron mobility greater than 20 000 cm^2^ V^–1^ s^–1^ at room temperature, compared to those of the more commonly used semiconductors, silicon and GaAs (1500 and 8500 cm^2^ V^–1^ s^–1^, respectively).^3,4^ This gives InAs a particular application within high frequency field effect transistors and magnetoresistive sensors.^4^

Owing to the importance of III/V semiconductors within the optoelectronics industry, much research has been directed towards their deposition.^5–7^ The dominant processes used for the fabrication of III/V materials are molecular beam epitaxy (MBE) and chemical vapour deposition (CVD).^8–10^ Whilst MBE is a useful tool for the development of novel materials, the scale of materials fabrication by this method is limited. CVD, in contrast, offers good potential for large-scale production of such materials. Additional advantages provided by CVD are the ease of fabrication and costs, since depositions are carried out at atmospheric pressure using molecular precursors. Furthermore, the robustness in terms of adhesion to the substrate of CVD prepared films are almost always superior to films deposited by physical vapour deposition (PVD).^11^ CVD also allows the growth of films with differing morphologies and grain sizes by simply changing the precursor, temperature or – in the case of Aerosol-Assisted CVD (AACVD) – the solvent.^12^ These are two factors that can greatly influence charge carrier mobility.

The majority of examples of III/V semiconductors by CVD employ dual precursors for the deposition of the binary material. This process is far from ideal given the extreme toxicity and high vapour pressure of arsine as a precursor and the pyrophoric nature of MMe_3_ (M = Al, Ga, In). Difficulties can also arise in control of film stoichiometry. As a result, there has been significant research interest in the development of III/V compounds containing preformed bonds between the desired elements (M–E, where M = group 13 metal and E = pnictogen) for potential application as single-source precursors to the relevant III/V material, bypassing the complications arising from the use of highly toxic precursors. Particular credit should be directed towards the work of Cowley and Jones with regard to the development of such precursors.^13^ Whilst much attention has been paid to the deposition of GaAs from single-source precursors by CVD processes, examples of the production of indium-based materials by such methods are much less common. The groups of Cowley and Bradley both independently reported the deposition of InP from the single-source precursor [Me_2_InPMe_2_]_2_ by metal organic (MO)CVD and MBE, respectively.^14–16^ In the latter case, indium-rich films were formed at all temperatures and the use of excess phosphine was required in the deposition process in order to achieve stoichiometric InP films. More recently, both InP and InAs were successfully deposited from the related dimeric organometallic species [^*n*^Bu_2_InE^*t*^Bu_2_]_2_ (E = P, As) *via* supercritical chemical fluid deposition, though the arsenide films were found to be poorly adherent to the substrate.^7^

Organometallic InAs compounds are a potentially useful source of InAs in deposition processes and this has driven the development of a range of complexes, taking the form of monomeric, oligomeric and cage structures. Simple monomeric adducts, such as [(^*t*^Bu)_3_In·As(SiMe_3_)_3_]^17^ and [(Me_3_SiCH_2_)_3_In·As(SiMe_3_)_3_]^18^ are seemingly a good starting point for such precursor chemistry, given their low molecular masses and correct stoichiometry. However, the application of such adducts is hindered by the weakness of the In–As dative interaction, in comparison to the σ-bond interactions of In–C and As–Si, which allow facile dissociation under elevated temperatures. Indeed, similar titanium and tin arsenide adducts yielded only TiO_2_ and SnO_2_, respectively, when used as precursors to the corresponding arsenides.^19,20^

Dimeric species of the form [R_2_InAsR′_2_]_2_ are the most common examples of potential InAs precursors. Higher oligomers have also been synthesised and structurally characterised, including the trimer [Me_2_InAsMe_2_]_3_,^21^ which exists as a six-membered ring, as well as a number of cage complexes which have been reviewed by Neumüller *et al.* ^22^ However, whilst a range of such complexes have been developed, few reports exist into their application as single-source precursors to InAs. The good solubility of such organometallic complexes in non-polar solvents offers the added potential for application in solution-based CVD processes, such as AACVD.^23,24^

AACVD is a low cost and scalable technique that has been used to produce high quality thin films for many optical and electrical applications and is an ideal technique to overcome the challenges of large-scale production of crystalline III–V semiconductor films. Recent research has highlighted the potential of single-source GaAs precursors and the technique of AACVD for the deposition of high quality GaAs thin films, suitable for application in photovoltaic devices.^25–27^ The AACVD technique relies on the solubility of the precursor and the requirement for volatility is removed.

Herein, the formation of a single-source precursor for the deposition of InAs thin films by AACVD is investigated *via* the methane elimination reaction between InMe_3_ and ^*t*^BuAsH_2_. The deposition of InAs *via* AACVD and electrical performance of the resulting thin films is reported for the first time.

## Experimental

### General procedures – synthesis

**Caution**! Trimethylindium and tertbutylarsine are pyrophoric substances that ignite spontaneously in air and therefore must be handled under an inert atmosphere. Tertbuylarsine is highly toxic and must be handled with care. All experiments must be carried out in a fume cupboard. Post deposition the films are air/moisture stable and safe to handle; all reactive species leave *via* the exhaust during the AACVD process.

Trimethylindium (99.999%) was used as received from SAFC Hitech Ltd. Tertbutylarsine (99.999%) was purchased and used as received from Dockweiler Chemicals. Indium arsenide (99%) was purchased and analysed as received from Alfa Aesar. All solvents were purchased from Alfa Aesar and dried over activated alumina by the Grubbs method using Anhydrous Engineering equipment, such that the water contamination was 5–10 ppm or less.^28^ Nitrogen (99.99%) was obtained from BOC and passed through activated molecular sieves to remove moisture.

### Precursor synthesis

Precursor synthesis was carried out in batch processes, stored as a stock solution in toluene (0.02 g mL^–1^) and used as required. The following describes a typical synthesis.

^*t*^BuAsH_2_ (1.00 g, 7.46 mmol) was cooled to –78 °C and dissolved in toluene (*ca.* 10 mL) and added dropwise to a stirring solution of InMe_3_ (1.00 g, 6.25 mmol) in toluene (*ca.* 10 mL) at –78 °C. The mixture was stirred at this temperature for *ca.* 30 min before being warmed slowly to room temperature, with which the slow evolution of gas was observed. The colourless reaction mixture was slowly warmed to 80 °C in an oil bath (during which time the evolution of gas was seen to increase) and allowed to stir for 16 hours. After this time, the evolution of gas had ceased and a yellow solution had formed, indicating the completion of the reaction. The solvent was removed *in vacuo* to yield a solid yellow product. The product was then redissolved in anhydrous toluene and stored as a stock solution of concentration 0.02 g mL^–1^ in a Youngs flask.

Crystals of the product were grown from a concentrated toluene solution, cooled to –20 °C. Yield = 0.21 g, 13%. ^1^H NMR *δ*/ppm (C_6_D_6_): 0.42–0.86 (30H, InMe), 1.31–1.62 (72H, As^*t*^Bu), 2.51–2.52 (2H, AsH). ^13^C{^1^H} NMR *δ*/ppm (C_6_D_6_): –5.2 to –2.0 (InMe), 31.3–40.4 (As^*t*^Bu). Anal. calcd for C_42_H_104_As_8_In_8_: C, 23.71; H, 4.93. Found: C, 24.24; H, 5.06. MS (*m*/*z*; found): 145 [InMe_2_].

### General procedures – AACVD

Films were grown on SiO_2_ barrier coated float-glass substrates supplied by Pilkington NSG Ltd, of dimensions *ca.* 90 mm × 45 mm × 4 mm. Prior to use, the substrates were cleaned using water and detergent, propan-2-ol and acetone and were dried in air. For each deposition, two substrates were placed within the reactor with the lower surface of the ‘top-plate’ sitting at a distance of *ca.* 8 mm above the upper surface of the ‘bottom-plate’. The reactor was heated to the relevant temperature by a graphite block containing a Whatman cartridge heater and the temperature of the block was monitored by a Pt–Rh thermocouple. All deposition temperatures quoted refer to this temperature. The temperature of the top-plate was generally found to be *ca.* 50–70 °C lower than that of the bottom-plate. After reaching the desired temperature, the temperature within the reactor was allowed to equilibrate for 30 min prior to commencing the deposition.

The precursors were dissolved in anhydrous toluene using standard Schlenk techniques, from which an aerosol was generated at room temperature by use of an ultrasonic humidifier. The aerosol was carried into the reactor using nitrogen through a brass baffle to obtain laminar flow. The total time for each deposition was dependent upon the solvents, volumes and flow rates being used. Depositions were carried out at substrate temperature of 450 °C.

### Analysis techniques

Thermogravimetric analysis was carried out on a Netzsch system from 20 to 600 °C. Samples of *ca.* 10–20 mg were run under an inert atmosphere of helium in sealed aluminium pans. X-ray diffraction (XRD) was used to analyse the samples in a modified Bruker-Axs D8 diffractometer with parallel beam optics equipped with a PSD LinxEye silicon strip detector to collect diffracted X-ray photons. This instrument uses a Cu source for X-ray generation with CuKα_1_ and CuKα_2_ radiation of wavelengths 1.54056 Å and 1.54439 Å respectively, emitted with an intensity ratio of 2 : 1, a voltage of 40 kV and current of 30 mA. The incident beam angle was kept at 1° and the angular range of the patterns collected was 10° < 2*θ* < 66° with a step size of 0.05° counted at 0.5 s per step.

Scanning electron microscopy (SEM) was performed to determine surface morphology and film thickness using a JEOL JSM-6301F Field Emission SEM at an accelerating voltage of 5 keV. Images were captured using SEMAfore software. Samples were cut to 10 mm × 10 mm coupons and coated with a fine layer of gold to avoid charging. X-ray photoemission spectroscopy (XPS) was performed using a Thermo Scientific K-alpha photoelectron spectrometer using monochromatic Al-K_α_ radiation. Survey scans were collected in the range 0–1100 eV (binding energy) at a pass energy of 160 eV. Higher resolution scans were recorded for the principal peaks of In (3d), As (3d), O (1s) and C (1s) at a pass energy of 50 eV. Peak positions were calibrated to carbon (284.5 eV) and plotted using the CasaXPS software. Energy dispersive (EDX) analysis was carried out on a JEOL JSM-6301F Field Emission instrument with acceleration voltage of 20 kV. The As atom% was derived from the As-K_α_ line (10.530 keV) and the In atom% derived from the In-L_α_ (3.286 keV).

Hall effect measurements were carried out on an Ecopia HMS-3000 Hall Measurement System using the van der Pauw method to determine the sheet resistance, free carrier concentration (*N*) and mobility (*μ*). A square array of ohmic contacts arranged on 1 cm^2^ samples were then subjected to an input current of 1 mA and a calibrated magnetic field of 0.58 T. The transverse voltage was then measured. The measurement was repeated by reversing the direction of the magnetic field and the current.

### Density functional theory

Density functional theory (DFT) within the Vienna *ab initio* Simulation Package (VASP)^29–32^ was employed to calculate the electronic structure of InAs. The screened hybrid HSE (Heyd–Scuseria–Ernzerhoff)^33^ exchange and correlation functional with 31% of exact Hartree Fock (HF) exchange was used. Hybrid functionals have proven to be more accurate in describing the electronic properties of materials and give a better electronic structure to standard functionals.^34–39^ To describe the interactions between the core (In:[Kr] and As:[Ar]) and the valence electrons, the Projector Augmented Wave Method (PAW)^40^ was used.

**Fig. 1:**
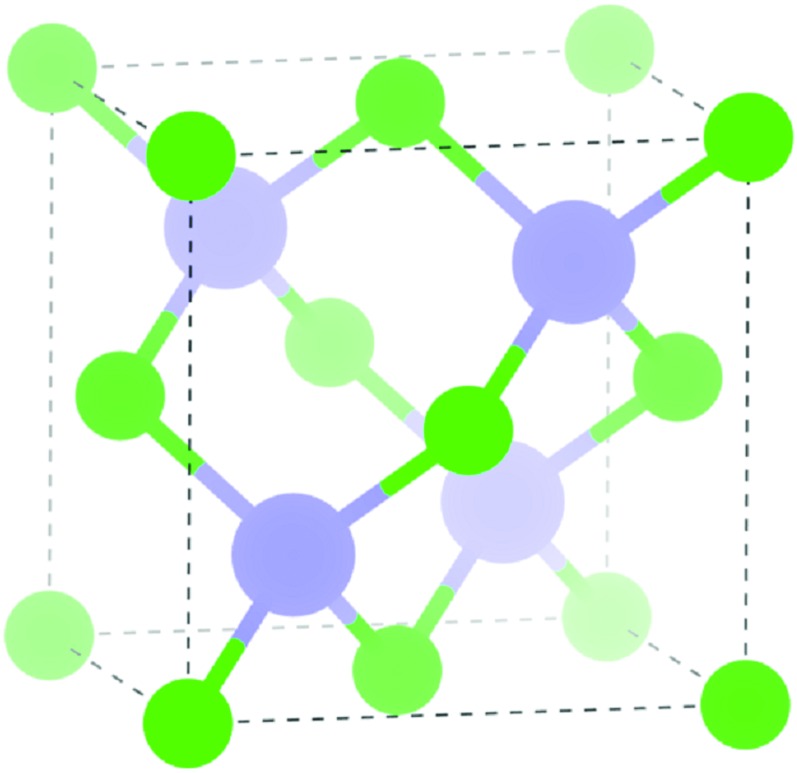
Unit cell of InAs in the zinc-blende structure, green = As, purple = In.

A *k*-point grid of *Γ*-centred 6 × 6 × 6 with a plane wave cut-off energy of 300 eV were found to be sufficient for convergence for the 2 atom cell of InAs (zinc-blende structure, *F*43*m* in Herman–Mauguin notation as shown in Fig. 1). A structural optimisation was carried out on the InAs structure where the cell volume, lattice vectors, cell angles and atomic positions were all allowed to relax. When the forces on all the atoms was less than 0.01 eV Å^–1^ the system was deemed to be converged. The structural optimisation was followed by a density of states (DOS) calculation and a band structure calculation, where spin–orbit coupling effects were taken into account.

### X-ray crystallography

Details of the crystallographic data collection and refinement are given in Table 1. Suitable crystals were selected and mounted on a nylon loop and the dataset was collected on a Rigaku AFC12 goniometer equipped with an enhanced sensitivity (HG) Saturn724 + detector mounted at the window of an FR-E + SuperBright molybdenum rotating anode generator (*λ* = 0.71073 Å) with VHF Varimax optics (100 mm focus) was used. Cell determination, data collection, data reduction, cell refinement and absorption correction were carried out by using CrystalClear-SM Expert 3.1 b18.^41^ Structure solution and refinement were carried out by using WinGX and software packages.^42^ H atoms attached to C atoms were placed in geometrically assigned positions, with C–H distances of 0.98 Å (CH_3_) and refined by using a riding model, with *U*_iso_(H) = 1.5 *U*_eq_(C) (CH_3_). enCIFer was used to prepare CIFs for publication.^43^

**Table 1:** Crystal data and structure refinement for InAs precursor complex

Molecular formula	C_42_H_104_As_8_In_8_·2(C_7_H_8_)
*M* [g mol^–1^]	2311.43
*T* [K]	100(2)
Crystal system	Monoclinic
Space group	*P*21/*c*
*a* [Å]	14.4602(10)
*b* [Å]	12.4339(9)
*c* [Å]	23.7507(17)
*α*	90°
*β*	107.564(1)°
*γ*	90°
*U* [Å^3^]	4071.2(5)
*Z*	2
*F*(000)	2224
Total reflections	43 045
Unique reflections	9286
*R*_int_	0.0515
GooF on *F* ^2^	1.050
Final *R* indices [*I* > 2*σ*(*I*)]	*R*_1_ = 0.0324, w*R*_2_ = 0.0678
*R* indices (all data)	*R*_1_ = 0.0482, w*R*_2_ = 0.0749

## Results and discussion

### Precursor synthesis

With a view to synthesising a single-source InAs precursor, trimethylindium was reacted with a slight excess of *tert*-butylarsine in toluene. *Tert*-butylarsine was chosen as an arsenic source owing to its wide use in the deposition of arsenide materials and low pyrolysis temperatures.^15,44,45^ Both molecular dynamics studies and partial pressure analysis suggest a combination of free radical and β-elimination mechanisms in the decomposition process.^45,46^

**Fig. 2:**
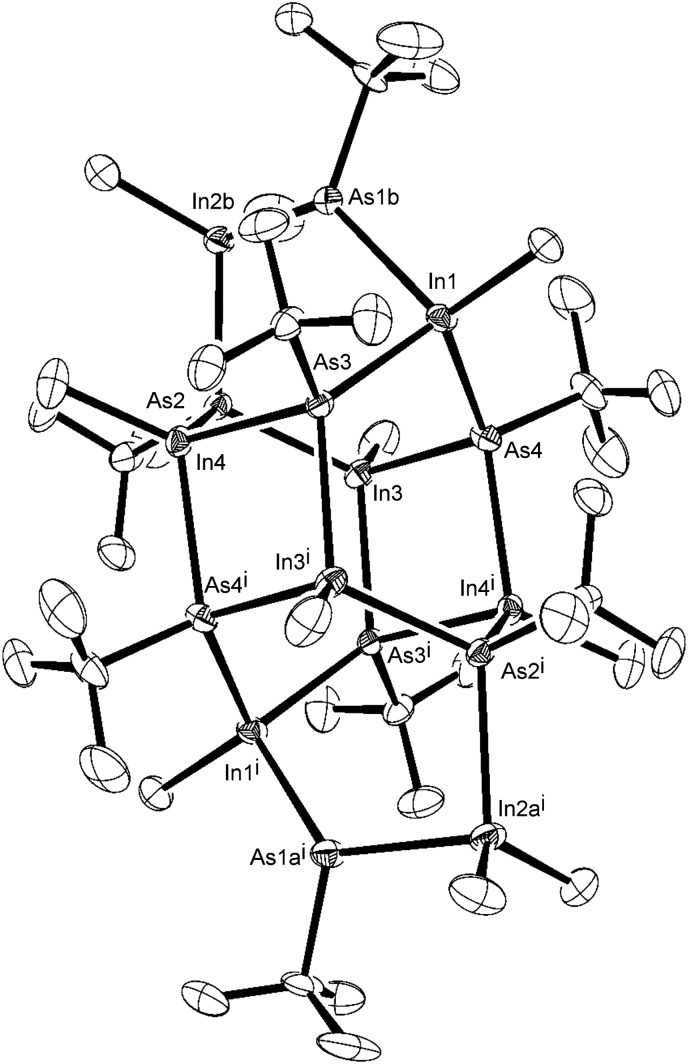
X-ray crystal structure of the indium arsenide cluster isolated from the methane elimination reaction between InMe_3_ and ^*t*^BuAsH_2_.

The ^1^H and ^13^C{^1^H} NMR spectra of the crude product from the reaction of InMe_3_ and ^*t*^BuAsH_2_, showed a number of resonances in the InMe, As^*t*^Bu and AsH regions, suggesting either the formation of an oligomeric species, or alternatively the formation of a number of different complexes. This indicated that the reaction did not stop as a simple Lewis acid–Lewis base adduct. Resonances in the ^1^H NMR spectrum in the region 0.42–0.86 ppm were attributed to InMe, those in region 1.31–1.62 ppm to As^*t*^Bu and two peaks at 2.51 and 2.52 ppm to AsH. Likewise, the ^13^C{^1^H} NMR spectrum showed resonances in the region –5.2 to –2.0 ppm (InMe) and 31.3 to 40.4 ppm (As^*t*^Bu). These resonances were all in agreement with NMR data for related InAs clusters.^7,47^ Recrystallisation of the crude product yielded yellow, rod-like crystals, which were suitable for single crystal X-ray diffraction and confirmed the formation of the cluster, [{(MeInAs^*t*^Bu)_3_}_2_(Me_2_InAs(^*t*^Bu)H)_2_], shown in Fig. 2 (hydrogen atoms omitted for clarity). Selected bond lengths and angles for the cluster are given in Table S1 (ESI†).

**Fig. 3:**
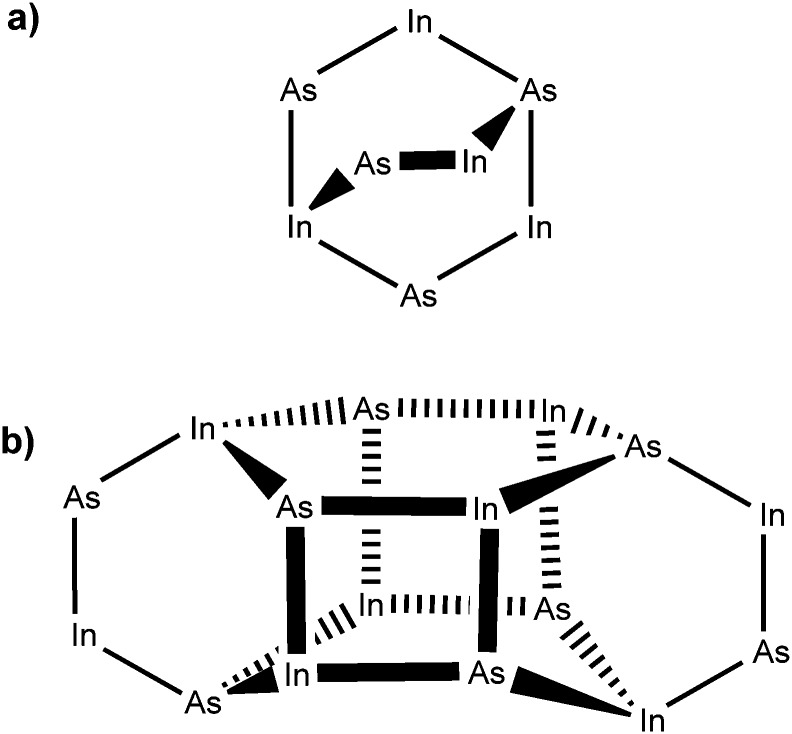
Structural representation of the oligomeric indium arsenide precursor complex, showing (a) an isolated eight-membered unit; and (b) the two interconnected units.

The centrosymmetric InAs cluster crystallised into the monoclinic space group *P*2_1_/*c*. Fig. 3 shows a simplified diagram of the complex with alkyl and hydride groups omitted for clarity. The structure can be considered as two conjoined units containing eight In/As centres (Fig. 3(a)). Each unit consists of a six-membered In–As ring in a distorted boat conformation, with each atom being four-coordinate, possessing three bonds to an adjacent As or In atom, and one further bond to either a methyl (In) or *tert*-butyl (As) group. Each ring is arched by one [–In(Me)_2_–As(^*t*^Bu)H–] bridge. The two major units are linked by four In–As bonds between the two rings (Fig. 3(b)) forming two additional In–As four-membered rings.

Whilst this particular oligomer was isolated and structurally characterised, it is unlikely that it was the only oligomer formed. Indeed, in solution it is likely that a number of oligomers existed in equilibrium, as would be suggested by the number of peaks present in the NMR spectra of the crystallised product. However, integration of peaks in the InMe, As^*t*^Bu and AsH regions of the ^1^H NMR spectrum were in a ratio of 15 : 36 : 1, consistent with the formation of the above complex. Furthermore, elemental analysis showed carbon to be present at 24.2% and hydrogen at 5.1%, in good agreement with the calculated values of 23.7 and 4.9%, respectively. Mass spectrometry did not show the presence of the parent ion for the cluster, displaying instead a large degree of fragmentation. The only peak of significant intensity was observed at 145 *m*/*z*, corresponding to [InMe_2_]. Hence, the formation of a number of cluster complexes occurs during the synthetic procedure, which contain a number of In–As linkages but with an overall In to As ratio of 1. This presents the precursor as potentially suitable for the formation of stoichiometric InAs *via* thermal decomposition.

### Thermogravimetric analysis (TGA)

**Fig. 4:**
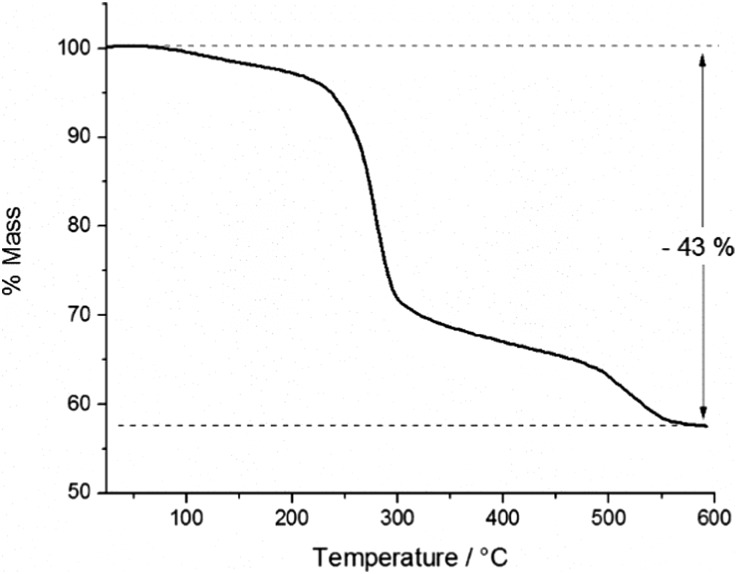
Thermogravimetric analysis of InAs precursor showing % mass loss with increasing temperature.

In order to study the thermal decomposition characteristics of the precursor, thermogravimetric analysis (TGA) was carried out on a 9.9 mg sample of the product (Fig. 4). A small initial mass loss of 3% was observed between *ca.* 70 and 200 °C, presumably due to sublimation of the sample.

The total mass loss incurred through the thermal decomposition was 43%, leaving a residual mass of 57% post-decomposition. Within any oligomer of the empirical formula [Me_2_InAs(H)^*t*^Bu]_*x*_, InAs contributes 68% of the mass, whilst in the cluster [{(MeInAs^*t*^Bu)_3_}_2_(Me_2_InAs(^*t*^Bu)H)_2_], InAs contributes 71% of the total mass of the complex. Indeed, for any of the likely products formed, the total InAs content can be said to be *ca.* 70% of the total mass. Thus the expected mass loss on removal of the organic ligands would be *ca.* 30%, as opposed to 43%, which was observed through experiment. This surplus mass loss was attributed to sublimation of some of the precursor within the aluminium crucible prior to decomposition. However, in spite of this the TGA results suggested the potential of the precursor for the deposition of InAs by CVD at temperatures below 600 °C, since the mass loss observed was greater than 30% and thus sufficient for decomposition to InAs.

### Aerosol-assisted chemical vapour deposition (AACVD)

A toluene solution (30 mL) of the precursor (0.2 g) was used to deposit InAs thin films *via* AACVD at 450 °C on glass substrates. It is worth noting that the solvent does not take part in the AACVD reaction but merely acts as a transport vector. The films were reflective blue/grey colour and well adhered to the substrate, passing the Scotch™ tape test.

### XRD, SEM and XPS analysis

**Fig. 5:**
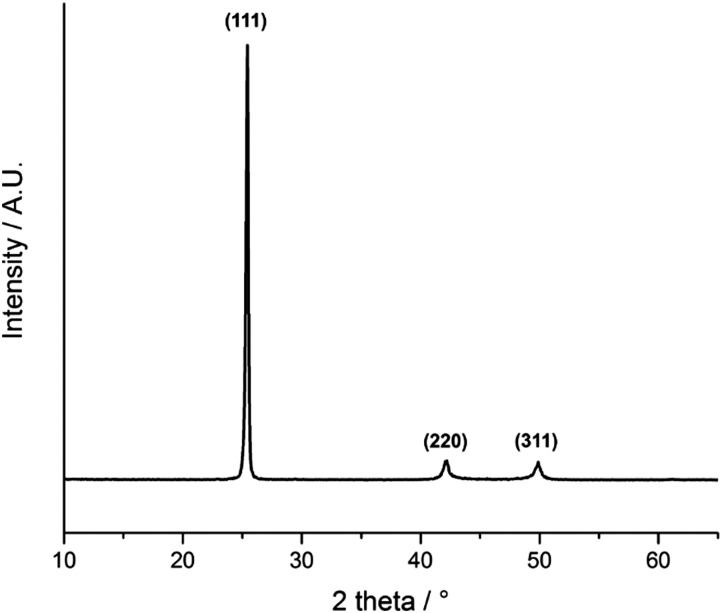
XRD pattern of InAs films grown from a toluene solution of the single source precursor at 450 °C *via* AACVD.

Fig. 5 shows the glancing angle powder X-ray diffraction (XRD) pattern of the AACVD deposited films. Peaks were observed at 2*θ* values of 25.4°, 42.2° and 49.9°, corresponding to the lattice planes (111), (220) and (311) of cubic InAs, respectively. The narrow peak width is evident of the high crystallinity of the films even at a deposition temperature of 450 °C. Preferred orientation was observed in the (111) plane most likely due to film growth taking place on an amorphous substrate.^48^

**Fig. 6:**
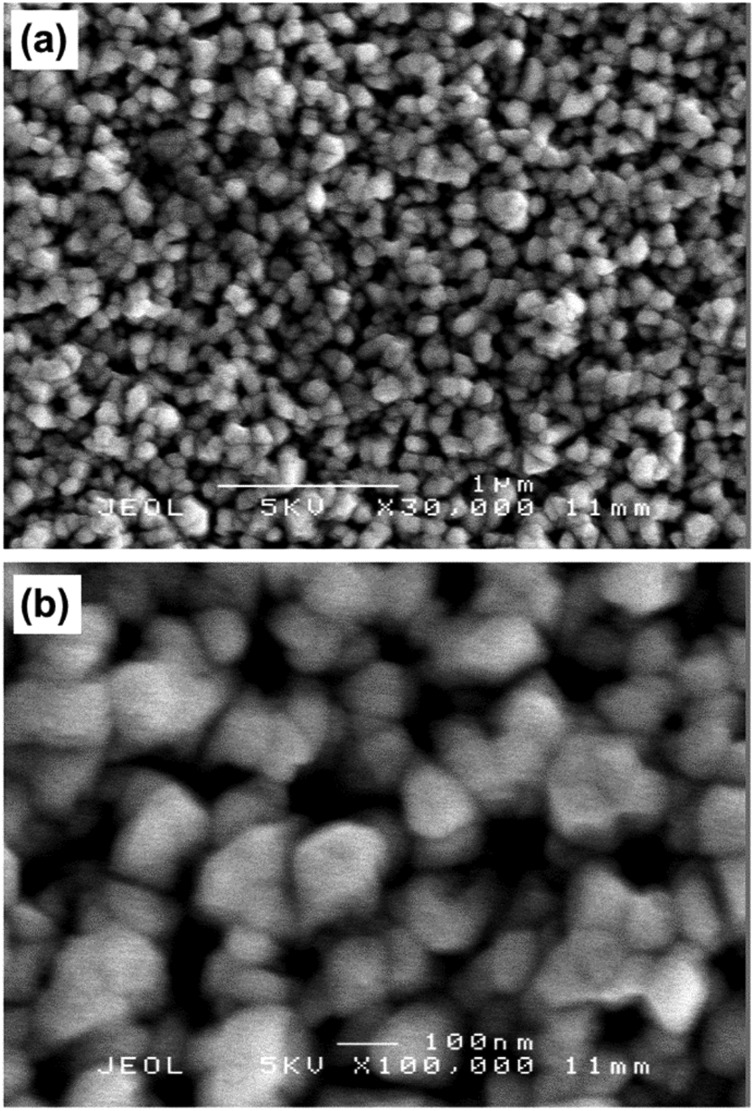
SEM images of InAs film grown *via* AACVD at 450 °C at (a) 30 000× and (b) 100 000× magnification.

The film microstructure was investigated by scanning electron microscopy (SEM), as shown in Fig. 6. The films were composed of fairly compact spherical features with grain sizes ranging between 100–500 nm. Side on SEM images indicated that the film thickness was *ca.* 500 nm (ESI,† Fig. S1).

Elemental analyses carried out using energy dispersive X-ray spectroscopy (EDX) showed that the films were stoichiometric with an In to As ratio of 1 : 1 (Table S2, ESI†). Core level X-ray photoelectron spectroscopy (XPS) data showed that the primary In 3d_5/2_ transition positioned at 444 eV corresponded to In bound to As and the primary As 3d_5/2_ transition at 40 eV matched transition for As in InAs (Fig. S2, ESI†).^49,50^ The presence of secondary 3d_5/2_ peaks for both In and As (445 and 44 eV, respectively)^51^ indicated the additional formation of native oxide species at the surface of the films, also confirmed in the O 1s regions of the spectra. The formation of the surface oxide is an expected observation for III/V materials with the rate of oxide formation of the metal arsenides decreasing in the order In > Al > Ga.^52^ This irreversible oxide formation has implications for semiconductor device performance, thus optimisation of the reaction conditions to minimise this effect is critical. Carbon-containing species were strictly limited to the surface of the films (*vide infra*).

To investigate the elemental composition within the bulk, XPS depth profiling in the In 3d_5/2_, As 3d_5/2_, O 1s and C 1s regions was carried out (Fig. S2, ESI†). Within the In 3d_5/2_ and As 3d_5/2_ regions, oxide species were only observed at the surface. Similarly, peaks in the O 1s region were no longer observed after etching. These observations indicated that oxide contamination was limited to the surface of the film and was therefore likely to have formed post-deposition. Carbon-containing species were also limited to the surface of the film, showing no retention of M–C bonds from the precursor.

### Electronic structure

**Fig. 7:**
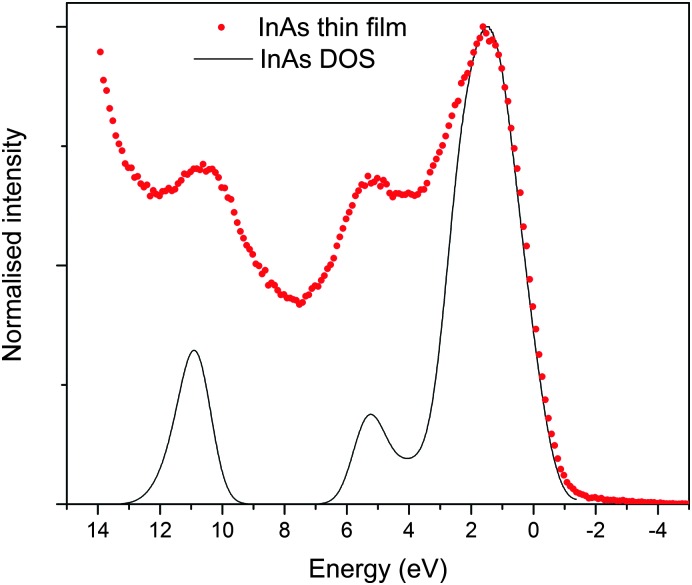
Normalized valence-band XPS spectrum of the InAs film grown *via* AACVD, superimposed onto simulated valence band XPS.

The valence band XPS taken of the sputter cleaned (to remove surface oxides) InAs film matched well with literature reports, standard pattern (also sputter cleaned, see ESI,† Fig. S3) and simulated spectrum.^53^ As shown in Fig. 7, the valence band is made up of three peaks centred at 1, 5 and 11 eV, these peaks originate from the outermost atomic s and p orbitals of In and As, *i.e.* 5s/5p and 4s/4p respectively.^53^ As observed by Ley *et al.*,^53^ the main peak in the valence band spectrum at 1 eV is relatively broad but appears to have some fine structure consisting of two features at the top of the peak and a shoulder at 3 eV. The band edge of the film and the InAs standard appeared close to 0 eV. This is as expected because at the surface of conventional n-type semiconductors the Fermi level lies close to the centre of the band gap and is lower relative to the bands edges than in the bulk of the material due to the presence of negatively charged acceptor-like surface states.^25,54^

**Fig. 8:**
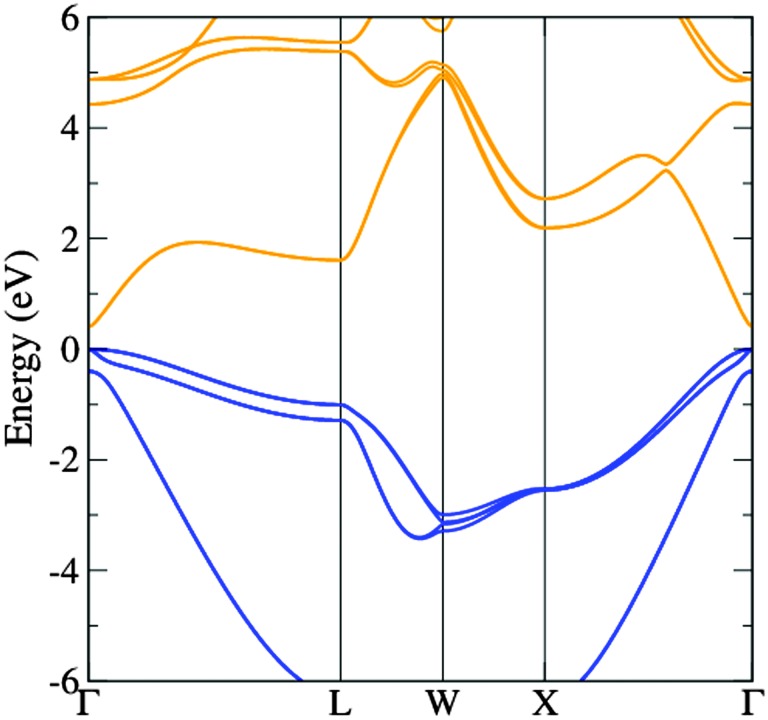
The calculated band structure of InAs showing the valence bands (blue) and the conduction bands (orange). The highest occupied state is set to 0 eV.

The band structure diagram shown in Fig. 8 displays the fundamental band gap between the valence band maximum (VBM) and the conduction band minimum (CBM) which is calculated to be 0.42 eV with a spin orbit splitting energy (ΔSO) of 0.39 eV, consistent with low energy experimental data taken from Madelung (0.42 eV and 0.38 eV respectively).^55,56^ The band structure shows excellent CB dispersion at the *Γ*-point with an effective electron mass of ∼0.03 me and a VB effective hole mass of ∼0.63 me at the *Γ*-point. This compares well to experimental values of ∼0.03 for the effective electron masses and 0.57 me for the effective hole masses.^57^ The calculated DOS was weighted and scaled using atomic orbital photoionisation cross-sections by Yeh and Lindau^58^ to form a simulated valence band XPS using a 0.47 eV Gaussian function to match experimental broadening. This was shown to be in excellent agreement with the experimental valence band XPS shown in Fig. 7.

### Electrical properties

The electrical properties of the as-deposited films were assessed through Hall effect measurements. The results showed the films to have n-type conductivity with a resistivity of 3.6 × 10^–3^ Ω cm, a bulk carrier concentration of 4.2 × 10^–18^ cm^–3^ and carrier mobility of 410 cm^2^ V^–1^ s^–1^. The electrical properties of the AACVD prepared InAs films look promising considering the depositions were carried out on amorphous glass substrates as opposed to lattice matched GaAs substrates, which is almost always reported in literature.^3,59^ It is expected that the electron mobilities can be further improved by optimisation of the deposition conditions and/or inclusion of post-deposition annealing steps, which will be the subject of future research.

## Conclusions

An investigation into the synthesis of a novel single-source precursor for the deposition of InAs *via* the methane elimination reaction between InMe_3_ and ^*t*^BuAsH_2_ has been described. Characterisation of the reaction product revealed the formation of an InAs cluster, which was subsequently utilised in the deposition of InAs thin films *via* AACVD. Hall effect measurements showed the films to be n-type semiconductors, however carrier mobilities were lower than expected for InAs, which characteristically shows high electron mobility. This was attributed to the presence of In_2_O_3_ and further deposition optimisation would be required to yield films with enhanced electrical properties.

The synthesis of a single-source precursor has been shown to be a useful route to the deposition of InAs thin films and the preformation of In–As bonds within the precursor was found to be critical to successful film formation. Furthermore, precursor synthesis can be carried out *in situ*, which would eliminate the need for prior isolation of the precursor and simplifying the overall deposition process. Whilst the carrier mobilities reported in this work were lower than required for device-quality thin films, the results described are the first example of the deposition of InAs by AACVD and highlight the facility and efficacy of the single-source precursor approach to the deposition of optoelectronic materials. Furthermore, comparison of experimental XPS valence band results with simulated electronic band structure showed excellent agreement.
