# Supplementary material for: A single-source precursor approach to solution processed indium arsenide thin films
Source: J Mater Chem C Mater. 2016 Jun 23;4(28):6761–8. doi: 10.1039/c6tc02293f (PMC5059786; doi:10.1039/c6tc02293f)
Supplement: Supplementary file 1 [file TC-004-C6TC02293F-s001.pdf]

Electronic Supplementary Material (ESI) for Journal of Materials Chemistry C.
This journal is © The Royal Society of Chemistry 2016

Supplementary information for manuscript 'A Single-Source Precursor Approach to Solution Processed Indium Arsenide Thin Films.'

Table S1. Selected bond lengths (Å) and angles (°) for [{(MeInAs^{t}Bu)$_3$}$_2$(Me$_2$InAs(^{t}Bu)H)$_2$]. Symmetry operation: #1 2 – x, 2 – y, 2 – z.

Selected Bond Lengths			
As(1B)-In(1)	2.6642(10)	As(2)-In(3)	2.6218(6)
As(1B)-In(2B)	2.684(2)	As(2)-In(4)	2.6337(5)
As(3)-In(1)	2.6202(5)	As(3)-In(3)#1	2.6655(5)
As(3)-In(4)	2.6587(5)	As(4)-In(4)#1	2.6835(5)
As(4)-In(1)	2.6327(6)	In(3)-As(3)#1	2.6653(5)
As(4)-In(3)	2.6595(5)	In(4)-As(4)#1	2.6836(5)
As(2)-In(2B)	2.680(2)		
C(1)-In(1)	2.175(4)	C(5)-In(4)	2.179(5)
C(2B)-In(2B)	2.178(10)	C(6B)-As(1B)	2.036(11)
C(3B)-In(2B)	2.176(8)	C(14)-As(3)	2.023(5)
Selected Angles			
C(1)-In(1)-As(3)	118.32(13)	As(3)-In(1)-As(4)	102.871(16)
C(1)-In(1)-As(4)	118.01(14)	As(3)-In(1)-As(1B)	97.99(5)
C(1)-In(1)-As(1B)	109.54(13)	As(4)-In(1)-As(1B)	107.93(6)
C(10)-As(2)-In(3)	107.13(14)	In(3)-As(2)-In(4)	114.079(17)
C(10)-As(2)-In(4)	107.66(12)	In(3)-As(2)-In(2B)	104.81(9)
C(10)-As(2)-In(2B)	108.76(13)	In(4)-As(2)-In(2B)	114.11(7)

Table S2: Average EDX results for the InAs film grown via AACVD and InAs standard obtained from Alfa Aesar.

	**In / at.%**	**As / at.%**
InAs Film	55.7	44.3
Standard	56.2	43.9

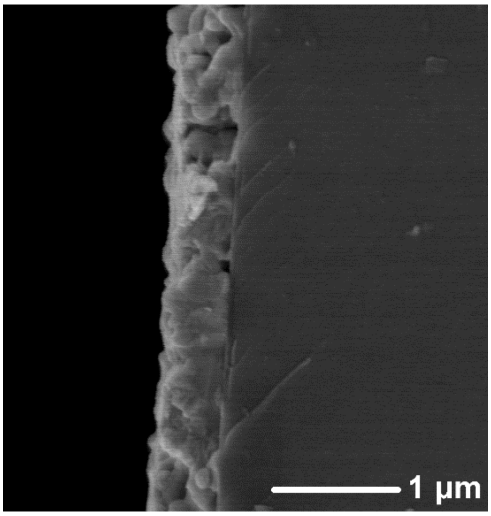

Figure S1 – Cross-sectional SEM images of InAs thin film deposited by AACVD.

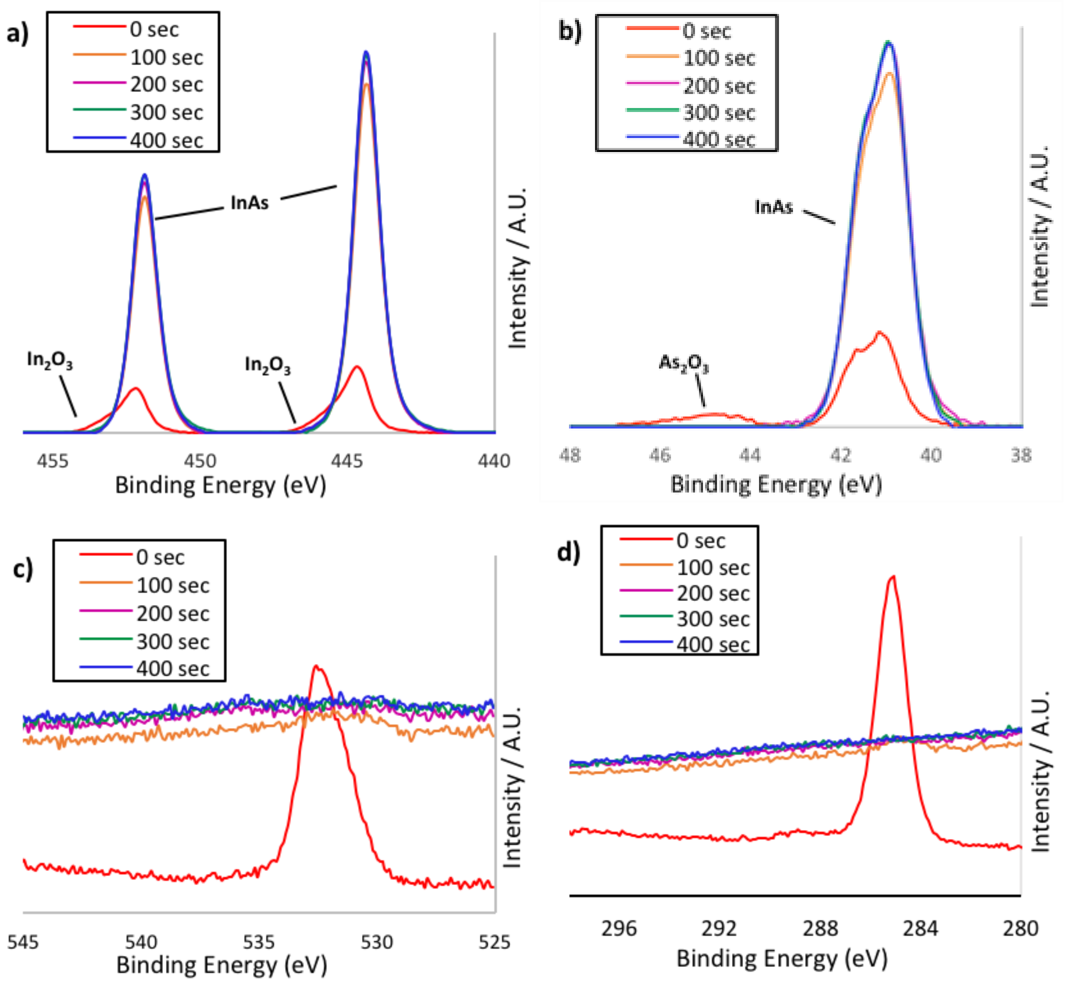

Figure S2. XPS depth profiling of the InAs film. a) In 3d peak; b) As 3d peak; c) O 1s peak; and d) C 1s peak.

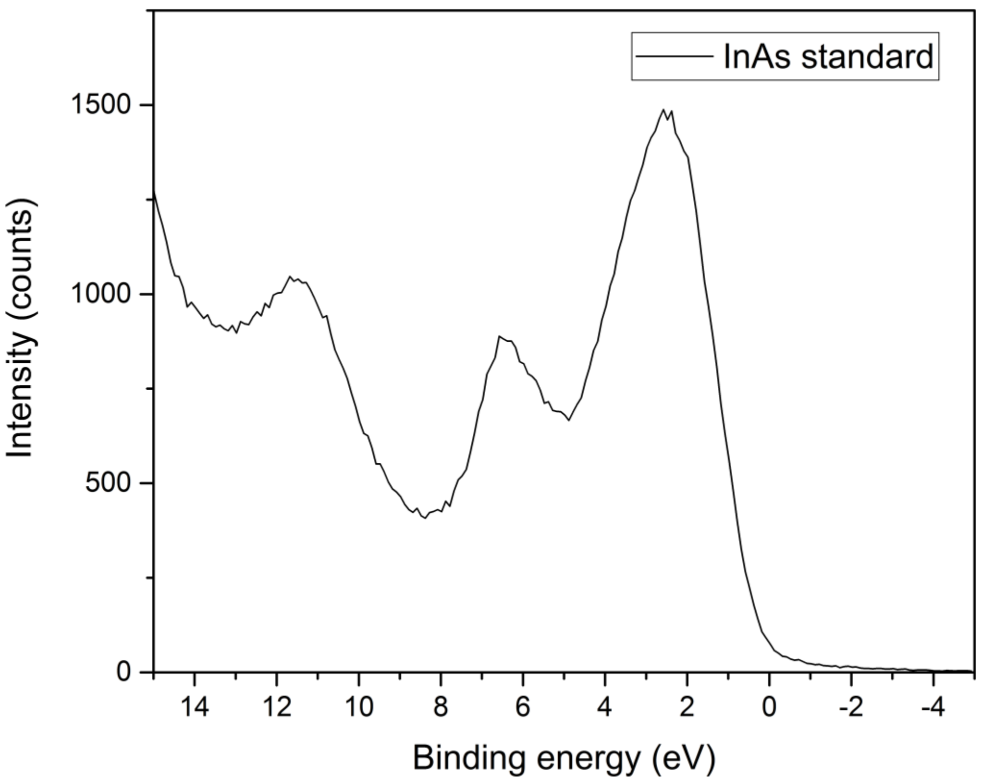

Figure S3. Valence band XPS of a) sputter cleaned InAs standard obtained from Alfa Aesar.
